# DEATH AND DYING IN GREEN CARE FARMS

**DOI:** 10.1093/geroni/igac059.941

**Published:** 2022-12-20

**Authors:** Judith Meijers, Kirsten Smit, Bram de Boer, Hilde Verbeek, Sascha Bolt

**Affiliations:** Maastricht University, Maastricht, Limburg, Netherlands; University Utrecht, Utrecht, Utrecht, Netherlands; Maastricht University, Maastricht, Limburg, Netherlands; Maastricht University, Maastricht, Limburg, Netherlands; Tilburg University, Tilburg, Noord-Brabant, Netherlands

## Abstract

Green care farms form an alternative to traditional nursing homes for people with dementia and combine agriculture production with health-related, social and educational services. Twenty-four-hour care green care farms offer end-of-life care. The aim of the study was to explore the experiences of healthcare workers and family caregivers with end-of-life care for people with dementia who died on a green care farm in the Netherlands. Semi-structured, in-depth interviews were conducted to explore their experiences with end-of-life care, including topics such as advance care planning, bereavement support and the influence of COVID-19. The study showed that most experiences were characterized by personal attention for the resident and family caregivers, and tailored holistic care. The duration of the dying phase was typically short, and most residents remained active until their final days. Despite the COVID-19 measures, healthcare workers and family caregivers still experienced adequate end-of-life care.

